# Bioaccessibility of phenolic compounds, lutein, and bioelements of preparations containing *Chlorella vulgaris* in artificial digestive juices

**DOI:** 10.1007/s10811-017-1357-2

**Published:** 2017-12-06

**Authors:** Bożena Muszyńska, Agata Krakowska, Jan Lazur, Barbara Jękot, Łukasz Zimmer, Agnieszka Szewczyk, Katarzyna Sułkowska-Ziaja, Ewa Poleszak, Włodzimierz Opoka

**Affiliations:** 1 Department of Pharmaceutical Botany, Faculty of Pharmacy, Jagiellonian University Medical College, Medyczna 9 St., 30-688 Kraków, Poland; 2 Department of Inorganic and Analytical Chemistry, Faculty of Pharmacy, Jagiellonian University Medical College, Medyczna 9 St., 30-688 Kraków, Poland; 3 Chair and Department of Applied Pharmacy, Medical University of Lublin, Chodźki 1, 20-093 Lublin, Poland

**Keywords:** *Chlorella vulgaris*, Dietary supplements, Lutein, Phenolic compounds, Artificial digestive juice

## Abstract

*Chlorella vulgaris* Beijerinck is a spherical, green alga belonging to the genus *Chlorella* and family Chlorellaceae. It has high nutritional value and shows multiple biological effects. Dietary supplements that contain extracts of *C. vulgaris* are sold in the form of tablets, capsules, powders, and aqueous solutions. To the best of our knowledge, this is the first study to determine the content of bioelements (zinc, iron, and magnesium), phenolic compounds, and lutein before and after incubation with artificial digestive juices from preparations containing *C. vulgaris*. In this study, we used commercial preparations in the form of powder and tablets. The samples were incubated in artificial gastric juice and then in artificial intestinal juice for 30 and 90 min. The contents of bioelements were determined by using the flame atomic absorption spectrometric method. Lutein and phenolic compounds were analyzed by high-pressure liquid chromatography. We also aimed to evaluate the quality of chlorella-containing formulations by using the methods described in the European Pharmacopoeia 8th edition. According to the results, the preparations containing *C. vulgaris* demonstrated the presence of phenolic compounds and lutein. Therefore, daily supplementation of preparations containing *C. vulgaris* substantiates its usefulness for humans. The qualitative composition of the examined organic substances and bioelements was found to be in accordance with the manufacturer’s declarations on the packaging containing *C. vulgaris* compared with the control samples; however, the contents of bioelements were found to be negligible after incubation with artificial digestive juices. This shows that the examined preparations containing *C. vulgaris* are not good sources of bioelements such as zinc, iron, or magnesium.

## Introduction

A sharp increase in the sales of nutritional supplements for particular uses and also an increase in over-the-counter (OTC) medicines have been observed in recent years. This can be attributed to the fast pace of life, and most of all, to the lack of time for people to follow the rules of a well-balanced diet. Therefore, there is considerable interest among people to balance their nutritional status with pharmacological sources of essential bioelements (e.g., zinc, iron, and magnesium) and biologically active substances taken in the form of readily available and assimilable preparations (e.g., tablets, powders, and syrups) distributed primarily through pharmacies. It is important to check and analyze not only the market of dietary supplements and the honesty of promises presented by the manufacturers in their advertisements but also the content of active ingredients in these preparations. Some of the OTC formulas, dietary supplements, and functional foods fulfilling the demand for most of the nutrients affect health contain algae. Preparations containing algae are available as ready-made preparations: powders—lyophilizates, tablets, pills, and capsules and are commonly used in the production of cosmetics (Görs et al. 2010).

*Chlorella vulgaris* Beijerinck is a spherical, single-celled freshwater alga from the Chlorellaceae. It contains numerous bioactive organic and inorganic substances that exhibit health promoting properties; for example, it is has antihypertensive, anti-inflammatory, antioxidant, anticancer, and immunostimulatory properties as well as it also improves brain function (Suetsuna and Chen 2001; Tokusoglu 2003; Terés et al. 2008; Seyfabadi et al. 2011; Přibyl et al. 2013).

Kwak et al. (2012) performed experiment on a group of 40 healthy volunteers and demonstrated the immunomodulatory effects of a *C. vulgaris* extract. According to their results, there was an increase in the cytotoxic activity of natural killer cells and an increase in the concentration of interferon-γ and interleukin-1β after 8-week administration of *C. vulgaris* extract in the form of tablets. Oral administration of aqueous extract of *C. vulgaris* in mice decreased the production of IgE-antibodies and simultaneously increased the mRNA expression of T helper cell cytokines, including interferon-γ and interleukin-12 (Hasegawa et al. 1999). The mechanism of anticancer activity of *C. vulgaris* extracts also involves the stimulation of production and maturation of granulocytes and macrophages (Justo et al. 2001).

Compounds that are responsible for the aforementioned biological activities are among others phenolic compounds, xanthophylls such as lutein, and bioelements such as zinc, iron, and magnesium. These substances are also specified by the manufacturers of *C. vulgaris* dietary supplements.

Phenolic compounds exhibit a wide spectrum of biological activities that are attributed to their strong antioxidant activity and have the ability to protect important cellular structures such as cell membranes, structural proteins, enzymes, membrane lipids, or nucleic acids against oxidative damage (Terpinc and Abramovic 2010). Phenolic compounds found in the methanolic extract of *C. vulgaris* may be responsible for its higher antioxidant activity (Aremu et al. 2016; Muszyńska et al. 2016).

It has been demonstrated that phenolic compounds found in *C. vulgaris* prevent the activity of free radicals thereby preventing the peroxidation of cell membranes of liver cells. This indicates that *C. vulgaris* has hepatoprotective activity (Peng et al. 2009). Phenolic compounds from *C. vulgaris* show potential antioxidant activity by neutralizing free radicals and prevent DNA damage, which in turn prevents tumorigenesis. Furthermore, the extracts of *C. vulgaris* activate apoptosis in tumor cells. Yusof et al. (2010) demonstrated the in vitro antitumor activity using HepG2 hepatocellular carcinoma cells after incubating the cells with extracts of *C. vulgaris* obtained using a hot method. Their results showed an increased expression of proteins such as p53 (transcription factor regulating the activation of DNA repair mechanisms and apoptosis in response to DNA damage), enhanced activity of Bax (proteins affecting an accelerated rate of the apoptosis process), caspase-3, and decrease in production of Bcl-2 proteins (B-cell lymphoma 2) that accelerate the apoptosis of tumor cells (Yusof et al. 2010). Naturally occurring lutein is produced primarily in higher plants and algae. Lutein is an important compound with antioxidant activity found in *C. vulgaris* and is essential for humans (Koushan et al. 2013). Lutein is the intracellular product of *C. vulgaris*, and thus lutein-rich *Chlorella* may be developed as a high-value health food (Shi et al. 1997).

In this study, we aimed to determine the content of bioelements because of their physiological role (in human metabolism by building blocks and being enzymes activators). Zinc is responsible for growth and proper functioning of the immune system (Livingstone 2015). Iron is an essential element in cellular aerobic respiration. Magnesium is the second most abundant intracellular cation and is an essential element responsible for maintenance of life. It is involved in various cellular functions and enzymatic reactions (Baaij 2015). Because of their high nutritional value and multiple beneficiary effects, dietary supplements containing *C. vulgaris* extracts are available in the market in the form of tablets, capsules, powders, and aqueous solutions. Numerous studies have described the content of biologically active substances in dietary supplements of *C. vulgaris* (Seyfabadi et al. 2011; Koushan et al. 2013; Přibyl et al. 2013). However, to the best of our knowledge, this is the first study to determine the content of bioelements (zinc, iron, and magnesium), phenolic compounds, and lutein in preparations containing *C. vulgaris* after incubation with artificial digestive juices (under conditions that stimulate the human gastrointestinal tract) which demonstrate their bioavailability. The secondary aim was to evaluate the quality of chlorella-containing formulations by using the methods described in the European Pharmacopoeia 8th edition ( 2013).

## Materials and methods

### Materials

Dietary supplements containing *Chlorella vulgaris* from a commercial origin, two preparations in the powdered form and four in the tablet form, were evaluated (Table 1). Names of the dietary supplements were changed to Chlorella S, A, O, M, B, and C to retain privacy.

**Table 1:** Dietary supplements containing *Chlorella vulgaris* which were used in study

Product preparation	Form	Expiry date	Country
Chlorella S	Powder	11. 2017	Poland
Chlorella A	Powder	07. 2018	China
Chlorella O	Tablets	10. 2018	China
Chlorella M	Tablets	08. 2018	China
Chlorella B	Tablets	03. 2018	China
Chlorella C	Tablets	01. 2019	Taiwan
Chlorella C	Tablets	09. 2017	Taiwan

### Reagents

All phenolic compounds used in this study were of standard high-pressure liquid chromatography (HPLC) grade. *p*-Coumaric was from Fluka (Switzerland) and *p*-hydroxybenzoic acid, cinnamic acid, kaempferol*-*7rhamnoside, apigenin, and the xanthophyll lutein were purchased from Sigma-Aldrich (USA). Epigallocatechin and epigallocatechin gallate were from ChromaDex (USA).

HPLC grade methanol, acetic acid, dichloromethane, and petroleum ether were from Merck (Germany). MgCl_2_ was from Chempur (Poland); NaCl, KCl, and NaHCO_3_ were from PPH Golpharm (Poland); pepsin and bile salts were from BTL (Łódź, Poland); CaCl_2_ was from Pharma Zentrale GmbH (Germany); pancreatic extract, HCl, KCl, concentrated HNO_3_ Suprapur, and KNO_3_, Suprapur were from Merck (Germany); C_6_H_8_O_7_, ZnSO_4_, KHCO_3_, Na_2_HPO_4_, K_2_HPO_4_, and NaOH were from the Polish Company of Chemistry (Gliwice, Poland). Water (quadruple-distilled) with a conductivity of less than 1 μS cm^−1^ was obtained using an S2-97A2 distillation apparatus (ChemLand, Poland).

### Preparation of artificial digestive juices

#### Artificial saliva

Briefly, 100 mL of KH_2_PO_4_ at a concentration of 25 mmol L^−1^, 100 mL of Na_2_HPO_4_ at a concentration of 24 mmol L^−1^, 100 mL of KHCO_3_ at a concentration of 150 mmol L^−1^, 100 mL of MgCl_2_ at a concentration of 1.5 mmol L^−1^, 6 mL of C_6_H_8_O_7_ at a concentration of 25 mmol L^−1^, and 100 mL of CaCl_2_ at a concentration of 15 mmol L^−1^ were subsequently added to a flask and then, four-time-distilled water was added to bring the total volume to 1000 mL (Arvidson and Johasson 1985).

#### Artificial gastric juice

Briefly, 2.0 g of NaCl and 3.2 g of pepsin were dissolved in four-time-distilled water; then, 80 mL of HCl at a concentration of 1 mol L^−1^ was added to bring the volume to 1 L (Polish Pharmacopoeia 2014).

#### Artificial intestinal juice

Briefly, 20 mg of the pancreatic extract, 120 mg of a bile salt, and 8.4 g of NaHCO_3_ were dissolved in four-time-distilled water to obtain a total volume of 1 L (Neumann et al. 2006).

### Apparatus

The release of active compounds from the preparations containing *C. vulgaris* was examined using the prototype Gastroel-2014 apparatus, which was constructed at the Department of Inorganic and Analytical Chemistry at the Faculty of Pharmacy, Medical College, Jagiellonian University (Opoka et al. 2016). This apparatus was used to examine the release of compounds into the artificial digestive juices; it imitates gastrointestinal motions and provides a constant temperature of 37 °C.

Mineralization of the preparations containing *C. vulgaris* was performed in the Magnum II microwave mineralizer ERTEC (Poland) for 1 h in three magnetron cycles: 15 min at 60% power, 15 min at 80% power, and 30 min at 100% power. Mineralization of solutions after digestion with artificial digestive juices using Gastroel-2014 was performed in the UV R-8 mineral Polish mineralizer, which was performed by UV irradiation of the mineralized test solution in a quartz reaction vessel in 5 cycles of 6–8 h each.

Thermo Scientific AA Spectrometer iCE 3000 SERIES UK was used to measure metals in samples.

The analysis of phenolic compounds and lutein was performed using an HPLC VWR Hitachi-Merck apparatus with the following analytical conditions: autosampler L-2200, pump L-2130, LiChrospher RP-18e column (250 × 4 mm, 5 μm) thermostated at 25 °C, column oven L-2350, and diode array detector L-2455 at the UV range of 200–400 nm.

### Sample preparation

#### Analysis of metals in the preparations containing *C. vulgaris*

The samples were mineralized to determine the content of metals (Mg, Zn, and Fe) in the preparations containing *C. vulgaris*. Then, 0.2 g of the preparations was weighed with an accuracy of 0.1 mg and was transferred into a Teflon vessel to which 2 mL of perhydrol and 6 mL of concentrated nitric acid were added. Mineralization was performed in a closed system (microwave mineralizer) until a clear, colorless solution was obtained. The solutions after mineralization were transferred to quartz evaporators and evaporated to “almost dry” on a heating plate at a temperature of approximately 200 °C to remove excess of reagents. Four-time-distilled water was added to the residue for a quantitative transfer to a volumetric flask, which was then filled with water to obtain a volume of 10 mL.

#### Analysis of metals and organic compounds in the extracts of preparations containing *C. vulgaris*

Extracts of *C. vulgaris* preparations were obtained as a result of in vitro digestion using Gastroel-2014. The samples were incubated with artificial gastric juice and in artificial intestinal juice for the same time intervals.

Initially, 0.5 g of each sample was weighed and transferred to 100 mL Erlenmeyer flasks which was then wetted with a solution of artificial saliva (2 mL, 1 min). To this, 100 mL of gastric juice was added and the flasks were closed with a stopper and placed in the apparatus. The process of incubation continued for the next 30 and 90 min. Then, the contents of the flasks were filtered using a Büchner funnel and a vacuum set. The residue was transferred to the Erlenmeyer flasks together with the filter and then 100 mL of intestinal juice was added. The digestion process lasted 30 and 90 min and then the extracts were filtered again. Next, 5 mL of the obtained filtrates was collected for each of the determination of metal content and organic compounds. A control sample was prepared in the same manner without adding the preparation of *C. vulgaris*. The content of bioelements in the analyzed, mineralized samples was examined by flame atomic absorption spectrometry (F-AAS). Lutein and phenolic acids were analyzed by reversed phase high-pressure liquid chromatography (RP-HPLC).

### Analysis of Zn, Fe, and Mg content before and after incubation with artificial digestive juices by using the F-AAS method

Concentrations of Zn, Fe, and Mg were determined using the F-AAS method. Thermo Scientific AA Spectrometer iCE 3000 series was used for all the measurements. Each sample was analyzed in quadruplicate, and the results are presented as mean values. Satisfactory agreement between the determined and the certified element concentration values was achieved.

### RP-HPLC analysis of phenolic compounds

The extracts obtained from the digestive juices were analyzed for the contents of phenolic compounds by using the RP-HPLC method. These analyses were performed according to the procedure developed by Sułkowska-Ziaja et al. (2017). The analyses were performed at 25 °C, with a mobile phase consisting of A—methanol, B—methanol:0.5% acetic acid, 1:4 (*v*/*v*). The gradient was as follows: 100% B for 0–20 min; 100–80% B for 20–35 min; 80–60% B for 35–55 min; 60–0% B for 55–70 min; 0% B for 70–75 min; 0–100% B for 75–80 min; and 100% B for 80–90 min at a flow rate of 1 mL min^−1^, *λ* = 254 nm (phenolic acids and catechins) and *λ* = 370 nm (flavonoids). Phenolic compounds were quantified by measuring the peak area with reference to the standard curve derived from five concentrations (0.03–0.50 mg mL^−1^). A quantitative analysis of phenolic compounds was performed using a calibration curve assuming the linear size of the area under the peak and the concentration of the reference standard. The results were expressed in milligram per 100 g of dry weight (d.w.).

### RP-HPLC analysis of lutein

Lutein in artificial digestive juice extracts was separated and analyzed by using an RP18 column (4.6 × 250 mm, 5 μm) at 30 °C. The mobile phase consisted of solvent A: methanol:water, 80:20 (*v*/*v*) and solvent B: methanol:dichloromethane, 75:25 (*v*/*v*). The following gradient procedure was used: starting at sample injection, 20% B for 5 min, 20–60% B for 5 min, 60–100% B for 25 min, 100% B for 5 min, 100–20% B for 10 min, and 20% B for 10 min. The flow rate was 1.0 mL min^−1^ (Yuan et al. 2008). The comparison of UV spectra at *λ* = 450 nm and the retention times with the standard compound enabled the identification of the lutein present in the analyzed samples.

### Analysis of tablet properties

The tablets were evaluated as per the standard procedure described in the European Pharmacopoeia 8th edition (2013.) for uniformity of weight, hardness, friability, and disintegration time. Tablets were also tested for variation in thickness to determine any variability associated with the tablet press or the method of preparation.

Average weight of tablets was obtained according to pharmacopeia limits by weighing 20 randomly selected tablets on an analytical balance (OHAUS Adventurer Pro). Hardness was determined for at least ten tablets by using the Erweka TBH 20 hardness tester (Erweka GmbH) and adopting a minimum hardness of 40 N as the acceptance criterion. For each formula, friability was evaluated from the percentage weight loss of 20 tablets tumbled in a Erweka TAR 120 friabilator (Erweka GmbH, Hausenstamm, Germany) at 25 rpm for 4 min. The tablets were dedusted, and the loss in weight caused by fracture or abrasion was recorded as the percentage weight loss. Friability of less than 1% was considered acceptable. The respective disintegration times of the prepared tablets were measured in 900 mL

of purified water with disks at 37 °C by using an ERWEKA ZT 222 tester (Erweka GmbH). Six tablets were randomly selected from each formulation and were put into a basket rack. The disintegration time was recorded until all the fragments of the disintegrated tablet passed through the screen of the basket. For nonmodified tablets, the disintegration time should be no longer than 15 min. Thickness of the tablet was determined for 20 tablets by using a digital vernier caliper (0–150 mm).

### Statistical analysis

Values are presented as mean ± standard deviation (SD). All experiments were performed four times. Statistical analysis was performed using one-way ANOVA with the Tukey–Kramer post hoc method of multiple comparisons. *p* < 0.05 was accepted as the level of statistical significance. Chemometric tools were used to facilitate the analysis and interpretation of the data obtained in the experiment; these included the two main methods: cluster analysis (CA) and principal component analysis (PCA). CA enabled the identification of groups of similar objects (preparation with *C. vulgaris*) that were described by nine parameters (concentration of metals and organic compounds). PCA as a method of calculation allowed the reduction of the data size and demonstration of the correlations between the objects in a two-dimensional space. Calculations were performed using GraphPad InStat (USA) and Statgraphics Centurion XVII. Statistical significance was established at *p* < 0.05.

## Results

The preparations of *C. vulgaris* were incubated with artificial digestive juices (Gastroel-2014 apparatus) to estimate the actual quantities of bioelements, phenolic compounds, and lutein available to humans. The incubation was performed under conditions imitating those in the human gastrointestinal tract (temperature of 37 °C and movements mimicking peristalsis in the gastrointestinal tract).

The following phenolic compounds were determined using the RP-HPLC method after incubation with artificial digestive juices from the preparations containing *C. vulgaris*: *p*-hydroxybenzoic acid, *p*-coumaric acid, cinnamic acid, kaempferol 7-rhamnoside, epigallocatechin gallate, apigenin, and lutein from the xanthophyll group (Table 2).

**Table 2:** Content of phenolic compounds and lutein in the extracts of preparations containing *Chlorella vulgaris* after digestion with artificial digestive juices

Artificial juice	Gastric juice		Intestinal juice		Methanolic extract
Extract to artificial digestive juice	30	90	30	90	
	After incubation in artificial saliva		After incubation in artificial intestinal juice		
*p*-Hydroxybenzoic acid (mg (100 g)^−1^)					
Chlorella S (powder)	1.18 ± 0.12 ^a^	1.08 ± 0.08 ^a^	1.66 ± 0.06	1.81 ± 0.98 ^a^	0.37 ± 0.01 ^a^
Chlorella A (powder)	0.64 ± 0.06 ^a,b^	0.55 ± 0.08 ^a,b^	2.74 ± 0.68 ^b^	2.15 ± 0.22 ^b^	0.38 ± 0.00 ^b^
Chlorella O (tablets)	1.05 ± 0.18 ^b,c^	0.91 ± 0.30 ^c^	1.56 ± 0.34	0.68 ± 0.03 ^b^	0.87 ± 0.00 ^a,b,c^
Chlorella M (tablets)	0.50 ± 0.02 ^a,c^	1.25 ± 0.19 ^b,d^	1.35 ± 0.09	1.13 ± 0.55	0.57 ± 0.03 ^a,b,c,d^
Chlorella B (tablets)	0.51 ± 0.05 ^a,c^	0.56 ± 0.05 ^a,d^	2.15 ± 0.29	0.84 ± 0.14	0.34 ± 0.00 ^c.d^
Chlorella C (tablets)	0.39 ± 0.05 ^a,c^	0.42 ± 0.03 ^a,c,d^	0.86 ± 0.26 ^b^	0.40 ± 0.03 ^a^	0.31 ± 0.07 ^c,d^
*p*-Coumaric acid (mg (100 g)^−1^)					
Chlorella S (powder)	nd	nd	nd	nd	2.53 ± 0.11 ^a^
Chlorella A (powder)	nd	nd	nd	nd	1.87 ± 0.08 ^a,b^
Chlorella O (tablets)	nd	nd	0.42 ± 0.20 ^c^	0.31 ± 0.15 ^c^	4.48 ± 0.17 ^a,b,c^
Chlorella M (tablets)	nd	nd	0.27 ± 0.10 ^d^	1.15 ± 0.02 ^c^	2.19 ± 0.02 ^a,b,c,d^
Chlorella B (tablets)	nd	nd	0.28 ± 0.05 ^e^	nd	2.31 ± 0.03 ^b,c,e^
Chlorella C (tablets)	nd	nd	nd ^c,d,e^	nd	1.62 ± 0.07 ^a,c,d,e^
Cinnamic acid (mg (100 g)^−1^)					
Chlorella S (powder)	0.27 ± 0.03 ^a^	0.34 ± 0.05 ^a^	0.24 ± 0.01 ^a^	0.14 ± 0.02 ^a^	0.02 ± 0.01 ^a^
Chlorella A (powder)	0.12 ± 0.01 ^a,b^	0.15 ± 0.02 ^a^	0.18 ± 0.02 ^a,b^	0.17 ± 0.03 ^b^	0.08 ± 0.00 ^a,b^
Chlorella O (tablets)	0.08 ± 0.001 ^a,b,c^	0.10 ± 0.09 ^c^	0.19 ± 0.02 ^a,c^	0.08 ± 0.001 ^a,b,c^	0.10 ± 0.00 ^a,b,c^
Chlorella M (tablets)	0.09 ± 0.002 ^a,d^	0.11 ± 0.09 ^a^	0.06 ± 0.001 ^a,b,c^	0.05 ± 0.007 ^a,b,d^	0.07 ± 0.00 ^a,b.c,d^
Chlorella B (tablets)	0.06 ± 0.001 ^a,b^	0.05 ± 0.004 ^a^	0.03 ± 0.005 ^a,b,c^	0.05 ± 0.00 ^a,b,c,d^	0.07 ± 0.00 ^a,b,c,e^
Chlorella C (tablets)	0.03 ± 0.005 ^a,b,c,d^	0.04 ± 0.001 ^a^	0.06 ± 0.003 ^a,b,c^	0.04 ± 0.001 ^a,b^	0.09 ± 0.00 ^a,b,c,d,e^
Kaempferol 7-rhamnoside (mg (100 g)^−1^)					
Chlorella S (powder)	8.03 ± 0.71 ^a^	10.88 ± 0.53 ^a^	9.01 ± 1.96	6.44 ± 1.13 ^a^	1.25 ± 0.01 ^a^
Chlorella A (powder)	6.35 ± 1.00	6.41 ± 1.40 ^a^	11.78 ± 1.70 ^b^	13.45 ± 3.05 ^a,b^	1.17 ± 0.00 ^a,b^
Chlorella O (tablets)	5.92 ± 0.85	6.65 ± 0.44 ^a^	7.53 ± 1.14 ^b^	5.64 ± 0.76 ^b^	4.32 ± 0.02 ^a,b,c^
Chlorella M (tablets)	5.63 ± 0.95 ^a^	6.85 ± 1.30 ^a^	5.87 ± 0.77 ^b^	5.13 ± 0.37 ^b^	2.77 ± 0.00 ^a,b,c,d^
Chlorella B (tablets)	5.23 ± 0.56 ^a^	5.21 ± 0.63 ^a^	7.19 ± 0.48 ^b^	6.25 ± 0.51 ^b^	1.31 ± 0.00 ^a,b,c,d,e^
Chlorella C (tablets)	4.47 ± 0.47 ^a^	5.16 ± 0.33 ^a^	5.80±0.71 ^b^	4.30 ± 0.31 ^b^	1.35 ± 0.01 ^a,b,c,d,e^
Epigallocatechin (mg (100 g)^−1^)					
Chlorella S (powder)	15.75 ± 1.15 ^a^	12.47 ± 3.16 ^a^	25.59 ± 5.23 ^a^	5.18 ± 0.57 ^a^	20.90 ± 0.66
Chlorella A (powder)	15.80 ± 0.84 ^b^	13.52 ± 1.70 ^b^	19.35 ± 1.60 ^a^	50.41 ± 5.6.0 ^a,b^	20.09 ± 0.14 ^b^
Chlorella O (tablets)	8.65 ± 0.52 ^a,b^	12.60 ± 1.10 ^c^	18.63 ± 3.13 ^c^	31.60 ± 5.98 ^a,b,c^	13.41 ± 1.27 ^a,b,c^
Chlorella M (tablets)	9.34 ± 0.83 ^a,b^	11.91 ± 3.28	11.59 ± 3.08 ^a,d^	7.81 ± 2.87 ^b,c,d^	6.37 ± 0.54 ^a,b,c,d^
Chlorella B (tablets)	8.56 ± 1.00 ^a,b^	5.92 ± 1.51 ^a,b,c^	20.22 ± 1.56 ^d,e^	54.27 ± 5.38^a,c,de^	8.42 ± 0.16 ^a,b,c,e^
Chlorella C (tablets)	10.17 ± 2.30 ^a,b^	10.13 ± 0.64	6.26 ± 1.15 ^a,b,c,e^	nd	14.04 ± 2.33 ^a,b,d,e^
Epigallocatechin gallate (mg (100 g)^−1^)					
Chlorella S (powder)	nd	nd	nd	nd	1.14 ± 0.23 ^a^
Chlorella A (powder)	nd	nd	nd	nd	2.17 ± 0.02 ^a,b^
Chlorella O (tablets)	nd	nd	nd	nd	1.63 ± 0.00 ^a,b,c^
Chlorella M (tablets)	nd	nd	nd	nd	1.36 ± 0.01 ^b,c^
Chlorella B (tablets)	nd	nd	nd	nd	1.42 ± 0.01 ^a,b^
Chlorella C (tablets)	nd	nd	nd	nd	nd
Apigenin (mg (100 g)^−1^)					
Chlorella S (powder)	16.84 ± 1.89 ^a^	23.40 ± 1.36 ^a^	23.26 ± 4.91 ^a^	16.93 ± 2.46 ^a^	2.95 ± 0.05 ^a^
Chlorella A (powder)	15.81 ± 0.17	18.11 ± 3.12 ^a^	18.50 ± 0.73	21.91 ± 2.70 ^a,b^	3.30 ± 0.01 ^a,b^
Chlorella O (tablets)	16.52 ± 0.48	16.10 ± 0.05 ^a^	20.00 ± 1.21	14.71 ± 0.04 ^b^	2.97 ± 0.21 ^b,c^
Chlorella M (tablets)	14.81 ± 0.01	18.94 ± 2.46	16.83 ± 0.05 ^a^	14.73 ± 0.01 ^b^	nd ^a,b,c,d^
Chlorella B (tablets)	16.02 ± 1.46	16.08 ± 1.14 ^a^	19.81 ± 0.10	16.31 ± 0.68 ^b^	3.22 ± 0.00 ^d^
Chlorella C (tablets)	13.66 ± 1.13 ^a^	15.58 ± 0.16 ^a^	18.93 ± 0.26	13.04 ± 1.14 ^b^	3.33 ± 0.20 ^a,c,d^
Lutein (mg (100 g)^−1^)					
Chlorella S (powder)	nd	nd	nd	nd	867.80 ± 90.41 ^a^
Chlorella A (powder)	nd	nd	nd	nd	723.47 ± 60.82 ^a,b^
Chlorella O (tablets)	nd	nd	70.58 ± 5.23 ^c^	50.45 ± 0.04 ^c^	497.17 ± 39.11 ^a,b.c^
Chlorella M (tablets)	nd	58.33 ± 0.01	58.36 ± 0.04 ^c^	50.93 ± 0.31 ^d^	468.05 ± 0.71 ^a,b,d^
Chlorella B (tablets)	nd	nd	67.85 ± 0.10	nd	73.12 ± 7.71 ^a,b,c,d,e^
Chlorella C (tablets)	nd	nd	57.24 ± 8.19 ^c^	42.91 ± 3.31 ^c,d^	527.56 ± 53.31 ^a,b,e^

*n* = 3 in triplicate; the Tukey–Kramer test was used to reveal the differences between paired groups of elements in rows; the same superscript letters (a, b, c, d, and e) are marked for which the content differences are statistically significant (for *p* values < 0.05)

*nd* not detected

The highest amounts of phenolic compounds released into the artificial digestive juices as compared to the control samples (methanol extracts) were as follows: *p*-hydroxybenzoic acid, cinnamic acid, kaempferol 7-rhamnoside, and apigenin. With respect to *p*-hydroxybenzoic acid, the largest amounts for all preparations were extracted in the artificial intestinal juice after an extraction time of 30 min (0.86–2.74 mg (100 g)^−1^ d.w.). *p*-Coumaric acid was determined only in tablets, and its content was significantly lower (0.27–1.15 mg (100 g)^−1^ d.w.) than that of methanolic extracts (1.62–4.48 mg (100 g)^−1^ d.w.). Cinnamic acid (both in the artificial digestive juices and in each time interval) was found in similar contents, which ranged from 0.03 to 0.34 mg (100 g)^−1^ d.w. Lower levels of this metabolite was noted in methanolic extracts (0.02–0.1 mg (100 g)^−1^ d.w.). Apigenin and kaempferol 7-rhamnoside were determined in significantly higher quantities in any time variant; their concentrations were up to 15 times greater than in the control samples (methanol extracts from *C. vulgaris*-containing preparations). Epigallocatechin was extracted in higher quantities from the intestinal juice after 90 min in case of powder (Chlorella A) and tablets (Chlorella B), 50.41 and 54.27 mg (100 g)^−1^ d.w., respectively, which was approximately 2.5–56 times greater than in case of control (20.09 and 8.42 mg (100 g)^−1^ d.w., respectively). A phenolic compound that was not released into the artificial digestive juices, but is extracted in methanol, was epigallocatechin gallate (1.14–2.17 mg (100 g)^−1^ d.w.).

Lutein, the primary metabolite present in algae, was released into the artificial digestive juices only from the tablets ranging from 42.91 to 70.58 mg (100 g)^−1^ d.w.

**Fig. 1:**
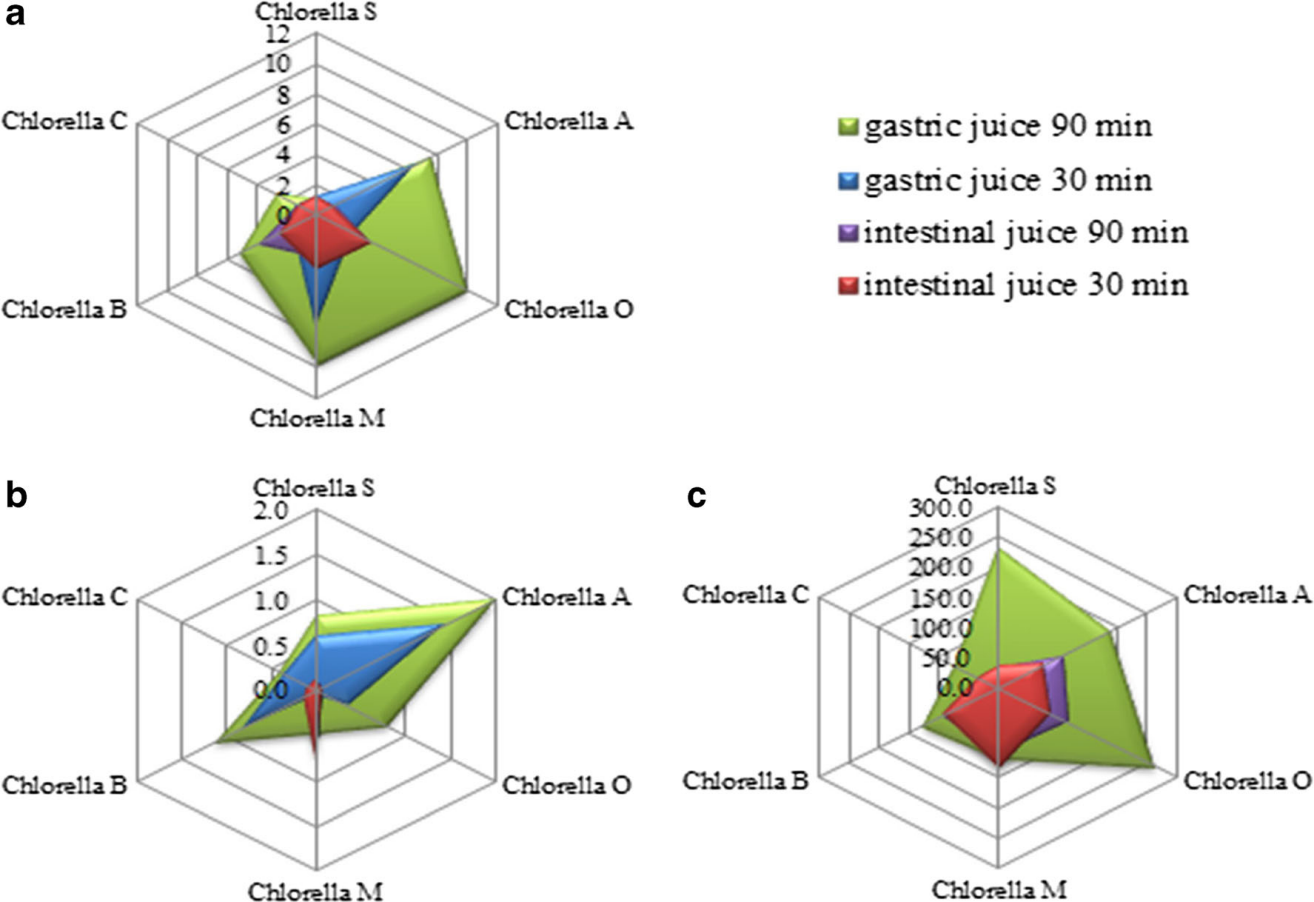
Radar chart showing the amount of extracted **a** Fe [μg g^−1^], **b** Zn [μg g^−1^], and **c** Mg [μg g^−1^] for artificial gastric juice and artificial intestinal juice, depending on the incubation time

F-AAS, which is one of the most common analytical techniques used to analyzed bioelements, was used to determine the content of Zn, Fe, and Mg metals in preparations containing *C. vulgaris* (powder and tablets) and in extracts obtained incubation with digestive juices. The developed mineralization conditions of lyophilized material and the applied analytical method allowed an effective determination of elements in the preparations and extracts of artificial digestive juices*.* In this study, the preparations containing *C. vulgaris* were subjected to quantitative determination of Zn, Mg, and Fe (Table 3). According to the literature, *C. vulgaris* is rich in macroelements such as phosphorus (1761.5 mg (100 g)^−1^ of dry matter), potassium (749.9 mg (100 g)^−1^), calcium (593.7 mg (100 g)^−1^), magnesium (344.3 mg (100 g)^−1^), and microelements such as iron (259.1 mg (100 g)^−1^) (Tokusoglu 2003). According to these data, the intake of 3 g of the *C. vulgaris* extract fulfills the daily iron requirements for men, whereas 7 g is needed for women (according to RDA standards, Institute of Medicine 2001) (Fig. 1a). According to the results of our study, the amount of iron found after incubation with artificial digestive juices is insufficient to supplement the deficiency of this element in humans, as in case of the release of zinc and magnesium from the preparations into the artificial digestive juices (Table 3). Thus, zinc content after digestion of samples for 30 min in artificial digestive juices (usually 9–15 tablets of these supplements are administered, corresponding to 4 g of extract per day) was found to be on an average only 0.804 μg, whereas the daily requirement of men and women is 11 mg (Fig. 1b). This implies that only 0.01% of the zinc requirement per day is supplied from the preparations containing *C. vulgaris*. *Chlorella vulgaris* contains chlorophyll, which constitutes 1–2% dry matter, and thus, it provides significant amounts of magnesium (Bashan et al. 2002). Magnesium content in control samples (mineralized formulations) was in the range of 1521–3221 μg g^−1^ d.w. However, after digestion with digestive juices, it was found to be much lower, and by using similar dosage assumptions as in case of zinc and magnesium, the average magnesium available was 306.57 μg (Fig. 1c). This quantity can only supplement 7.7% of the daily requirement for men and 9.9% for women. In case of iron, 8.74 μg was found in the sample after digestion with artificial digestive juices, which supplements 10.9% of the daily requirement for men and 4.8% for women.

**Table 3:** Content of bioelements determined using the F-AAS method in preparations containing *Chlorella vulgaris* after extraction to artificial digestive juices

Zn (μg g^−1^ of preparation)					
Digestive juice	Gastric juice		Intestinal juice		Control
Incubation time (min)	30	90	After 30 min in gastric juice	After 90 min in gastric juice	
Amount of Zn	Chlorella S (powder)				
released	0.58 ± 0.03 ^a^	0.81 ± 0.02 ^a^	0.13 ± 0.003 ^a^	0.11 ± 0.002 ^a^	8.25 ± 0.12 ^a^
	Chlorella A (powder)				
	1.43 ± 0.002 ^a,b^	1.99 ± 0.013 ^a,b^	0.03 ± 0.001 ^a,b^	0.05 ± 0.002 ^a,b^	28.94 ± 0.1 ^a,b^
	Chlorella O (tablets)				
	0.36 ± 0.002 ^a,b,c^	0.77 ± 0.008 ^a,b,c^	0.05 ± 0.001 ^a,c^	0.06 ± 0.001 ^a,b,c^	12.3 ± 0.1 ^a,b,c^
	Chlorella M (tablets)				
	0.074 ± 0.001^a,b,c,d^	0.48 ± 0.002 ^a,b,c,d^	0.80 ± 0.004 ^a,b,c,d^	0.67 ± 0.003 ^a,b,c,d^	9.4 ± 0.2 ^a,b,c,d^
	Chlorella B (tablets)				
	0.822 ± 0.003^a,b,c,d,e^	1.15 ± 0.002^a,b,c,d,e^	0.13 ± 0.001 ^b,c,d,e^	0.09 ± 0.001 ^a,b,c,d,^	48.1 ± 1.2 ^a,b,c,^
	Chlorella C (tablets)				
	0.367 ± 0.005^a,b,d,e^	0.42 ± 0.004^a,b,c,d,e^	0.01 ± 0.001 ^a,d,e^	0.09 ± 0.002 ^a,b,c,d,^	21.5 ± 0.3 ^a,b,c,^
Fe (μg g^−1^ of preparation)					
Amount of Fe released	Chlorella S (powder)				
	1.12 ± 0.02 ^a^	1.11 ± 0.02 ^a^	1.25 ± 0.03 ^a^	1.03 ± 0.03 ^a^	1210.1 ± 2.20 ^a^
	Chlorella A (powder)				
	6.58 ± 0.01 ^a,b^	7.54 ± 0.04 ^a,b^	1.14 ± 0.02 ^a,b^	1.23 ± 0.01 ^a,b^	1520.2 ± 4.5 ^a,b^
	Chlorella O (tablets)				
	2.44 ± 0.08 ^a,b,c^	9.96 ± 0.03 ^a,b,c^	3.60 ± 0.01 ^a,b,c^	3.15 ± 0.07 ^a,b,c^	2112.0 ± 2.0 ^a,b,c^
	Chlorella M (tablets)				
	7.24 ± 0.05 ^a,b,c,d^	9.81 ± 0.52 ^a,b,c,d^	3.48 ± 0.05 ^a,b,c,d^	2.79 ± 0.01 ^a,b,c,d^	1960.2 ± 5.0 ^a,b,c,d^
	Chlorella B (tablets)				
	1.54 ± 0.02 ^a,b,c,d,e^	5.12 ± 0.02 ^a,b,c,d,e^	2.42 ± 0.02 ^a,b,c,d,e^	3.90 ± 0.04 ^a,b,c,d,e^	1089.0 ± 7.5 ^a,b,c,d,e^
	Chlorella C (tablets)				
	1.33 ± 0.02 ^a,b,c,d,e^	2.82 ± 0.03 ^a,b,c,d,e^	1.22 ± 0.02 ^b,c,d,e^	1.35 ± 0.02 ^a,b,c,d,e^	879.6 ± 6.2 ^a,b,c,d,e^
Mg (μg g^−1^ of preparation)					
Amount of Mg released	Chlorella S (powder)				
	153.55 ± 1.66 ^a^	233.13 ± 4.60 ^a^	35.15 ± 2.63 ^a^	33.70 ± 1.20 ^a^	3221.5 ± 2.5 ^a^
	Chlorella A (powder)				
	239.82 ± 1.16 ^a,b^	188.18 ± 1.30 ^a,b^	77.30 ± 1.85 ^a,b^	105.92 ± 0.57 ^a,b^	1521.5 ± 4.0 ^a,b^
	Chlorella O (tablets)				
	93.80 ± 0.65 ^a,b,c^	262.68 ± 2.95 ^a,b,c^	87.91 ± 3.08 ^a,b,c^	115.28 ± 1.70 ^a,b,c^	2626.0 ± 5.3 ^a,b,c^
	Chlorella M (tablets)				
	117.31 ± 1.95 ^a,b,c,d^	117.34 ± 1.70^a,b,c,d^	133.43 ± 1.30 ^a,b,c,d^	108.15 ± 3.10 ^a,c,d^	1942.0 ± 3.1 ^a,b,c,d^
	Chlorella B (tablets)				
	58.47 ± 0.85 ^a,b,c,d,e^	125.74 ± 1.65^a,b,c,d,e^	92.16 ± 1.15 ^a,b,c,d,e^	92.84 ± 0.85^a,b,c,d,e^	2665.0 ± 6.0 ^a,b,c,d,e^
	Chlorella C (tablets)				
	50.77 ± 0.70 ^a,b,c,d,e^	68.91 ± 0.65 ^a,b,c,d,e^	33.91 ± 1.10 ^b,c,d,e^	24.05 ± 1.30^a,b,c,d,e^	3062.5 ± 2.1 ^a,b,c,d,e^

*n* = 3 in triplicate; the Tukey–Kramer test was used for revealing the differences between paired groups of elements in rows; the same superscript letters (a, b, c, d, and e) are marked for which the content differences are statistically significant (for *p* values of < 0.05)

Tablets containing *C. vulgaris* disintegrated in the artificial digestive juices only after approximately 30 min. Therefore, the physical properties of tablets containing *C. vulgaris* were ensured to be according to the European Pharmacopoeia 8th edition ( 2013). Table 4 lists the physical properties of the prepared tablets in terms of the uniformity of weight, hardness, friability, and disintegration time. The tablets were also tested for variation in thickness to determine any variability associated with the tablet press or the method of preparation.

**Table 4:** Evaluation of physical properties of tablets (mean ± SD) with *Chlorella vulgaris*

Formulations	Weight variation (mg)	Thickness (mm)	Friability (%)	Breaking force (N)	Disintegration time (min)
Chlorella O (tablets)	401.95 ± 15.72	5.78 ± 0.28	0.08	62.8 ± 8.3	42.16 ± 7.02
Chlorella M (tablets)	196.95 ± 2.31	4.17 ± 0.04	0.152	63.1 ± 5.93	36.67 ± 5.88
Chlorella B (tablets)	243.1 ± 3.32	4.3 ± 0.05	0.103	94.1 ± 7.13	40.17 ± 6.11
Chlorella C (tablets)	258.6 ± 6.35	5.06 ± 0.09	0.03	109.2 ± 16.94	125.67 ± 10.97

The thickness of the tablets ranged from 4.3 ± 0.05 to 5.78 ± 0.28 mm. In case of Chlorella B (tablets), the percentage deviation of thickness exceeded 5% (acceptable range of thickness: ± 5%). The average weight, hardness, and friability were within the pharmacopeia specifications. Variation in weight ranged from 196.95 ± 2.31 to 401.95 ± 15.72 mg (acceptable range of weight variation: ± 7.5% for tablets weighing up to 249 mg and ± 5% for tablets weighing more than 250 mg). Hardness of tablets ranged from 62.8 ± 8.3 to 109.2 ± 16.94 N (acceptable range of hardness: > 40 N), and friability ranged from 0.03 to 0.152% (acceptable range of friability: < 1%) (European Pharmacopoeia 8th edition, 2013). Disintegration times of the investigated tablets exceeded 15 min. All tablet formulations had excessively long disintegration times, which ranged from 36.67 to 125.67 min. The average disintegration time of the investigated tablets was up to 61.17 min.

## Discussion

The primary phenolic compounds determined in preparations containing *C. vulgaris* are both benzoic and cinnamic acid derivatives. According to the literature, *C. vulgaris* contains phenolic compounds such as salicylic acid, trans-cinnamic acid, chlorogenic acid, and caffeic acid (Miranda et al. 2001). In this study, we detected the presence of the following phenolic compounds in preparations containing *C. vulgaris*: *p*-hydroxybenzoic acid, *p*-coumaric acid, and cinnamic acid. In addition, the samples contained kaempferol 7-rhamnoside, epigallocatechin gallate, and apigenin. According to the literature, these compounds are bioavailable for humans.

*Chlorella* extracts rich in phenolic compounds exhibit strong antioxidant activity. Peng et al. (2009) performed in vivo experiments using rats to test the antioxidant activity of *Chlorella* extracts. The animals were fed on diet enriched with tetrachloromethane, an organic chemical compound in which all hydrogen atoms are replaced by strong, electronegative chlorine atoms exhibiting strong hepatotoxicity leading to jaundice and in severe cases to cirrhosis. Tetrachloromethane in the liver cells is metabolized to the trichloromethyl radical, which reacts with oxygen to form a more reactive radical –•CCl_3_O_2_•. According to their results, phenolic compounds found in *Chlorella* extracts prevented the damage caused by the free radical attack and the peroxidation of liver cell membranes, indicating the hepatoprotective activity of *Chlorella* extracts (Peng et al. 2009).

Lutein is a yellow organic carotenoid pigment. Its content in *C. vulgaris* ranged from 5 to 383 mg (100 g)^−1^ dry matter (Gonzalez and Bashan 2000; Kitada et al. 2009; Safi et al. 2014). In humans, the highest concentration of lutein is found in the yellow spot, which is a 7–8-mm round area on the inside of the retina with a distinctive yellow color due to the presence of lutein and zeaxanthin. Lutein exhibits a strong antioxidant effect on the free radicals of the reactive oxygen species (ROS) class, which are generated by oxidative phosphorylation in the mitochondria on the outer stamen segment. Furthermore, the increased production of ROS may be due to hypoxia of the photosensitive retinal cells (Koushan et al. 2013).

The amount of lutein, although less than in the control samples, were sufficient to supplement approximately 40–60% of the daily requirement for humans (the daily requirement ranges from 10 to 20 mg day^−1^) (Otten et al. 2006).

According to the results provided in Tables 2 and 3, six objects (preparations containing *C. vulgaris*) were found to be characterized by 11 features (variable with different concentrations of Mg, Zn, and Fe, and the content of the organic compounds). Large number of variables, whose values change over a relatively wide range, does not give a clear indication of the observations of systematic changes. In this case, the use of chemometric tools should be useful. Chemometric tools allow an analysis of data and their interpretation in an easy and accessible manner. The task of chemometry is to “extract” meaningful information about relationships between measured variables or objects. This is possible after applying appropriate mathematical analytical methods to eliminate the anomalies associated with the measurements (Johnson 1984; Sharaf et al. 1986; Miller and Miller 1999).

**Fig. 2:**
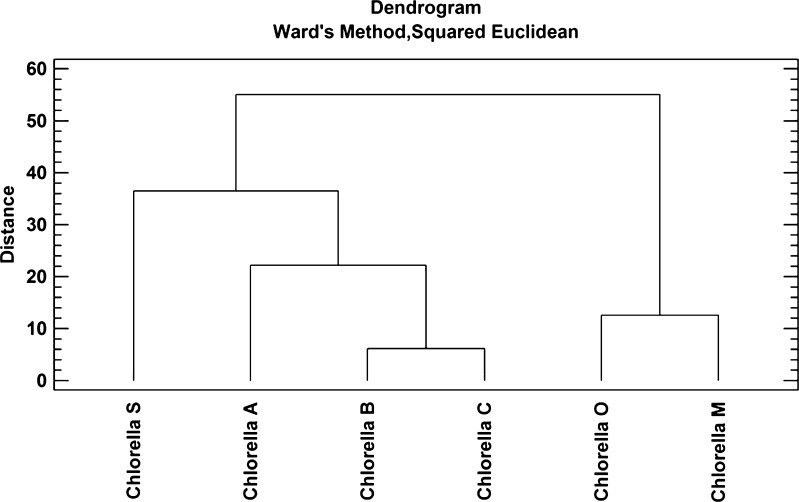
Cluster analysis of the preparation with *Chlorella vulgaris* (the Euclidean distance square and Ward’s algorithm)

CA was used for the first set of analytical dataset. This method makes it possible to indicate a similarity (presented on dendrogram commonly called “tree” (Fig. 2)), or a distinct absence, between the subject matter being investigated (preparations containing *C. vulgaris*) or variables (analyzed elements and organic compounds) (Aldenderfer and Blashfield 1985; Everitt et al. 2001; Massart and Vander 2004; Gemperline 2006).

Based on our similarity analysis (CA, Fig. 2), we observed a subgroup of parameters with similar variability. These parameters describe the concentrations of the individual variables. In practice, this implies that the course of change in these variables is similar, which proves at the same time, a high correlation between the objects. Thus, two primary aims were distinguished. Within the first cluster, there are four formulas: Chlorella S, Chlorella A, Chlorella B, and Chlorella C, and the second cluster has two formulas: Chlorella O and Chlorella M. Their classification into individual clusters indicates the similarity of their composition (content of organic constituents and metals). Furthermore, we found that the highest correlation occurred between Chlorella O and Chlorella M formulations as evidenced by the shortest length of the dendrogram tree arms. The shorter the branch lengths are, the greater is the similarity between the objects in question (Johnson 1984).

**Fig. 3:**
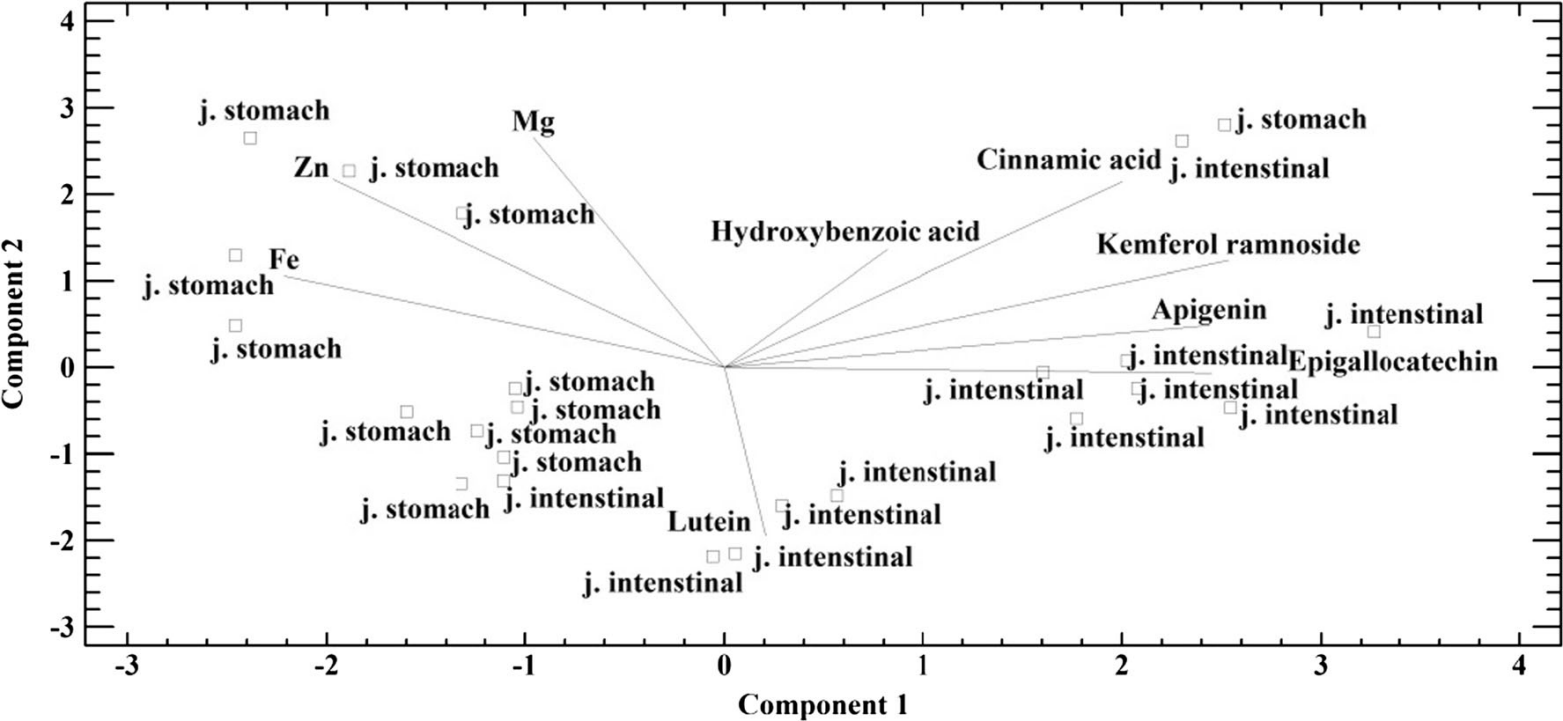
Biplot graph creates a three-dimensional space presented on the plane, showing the correlation between the analyzed ingredients found in the formulations containing *Chlorella vulgaris* and the site of the human gastric juice. Stomach juice (j. stomach) and intestinal juice (j. intestinal)

PCA has been used as the complementary method in this study. PCA is considered a computational method that leads to the limitation of the measurement data space to the amount required to describe the interactions between them. Parameters, mutually dependent, are replaced with new variables, the so-called main components, which exclude the loss of relevant information. Using PCA, we found that 75.4% of the variations occurring within the analyzed dataset could be described with the first three major components (PC1, PC2, and PC3). Bringing a multidimensional data system to the three main components allowed us to conduct the analysis on a flat projection of the three-dimensional space (Malinowski 1991; Henrion 1994; Massart and Vander 2004). Considering the similarity of the objects with respect to their place in the digestive system, which resulted in the release of metals and organic compounds from the formulations containing *C. vulgaris* (Fig. 3), two distinct groups were identified. The first group consisted of ingredients analyzed in the gastric juice, while the other group contained those analyzed in the intestinal juice. Such a division indicated the correlation between a given ingredient and the site of its release in the body. Furthermore, taking into account the two-dimensional graph (Fig. 3) obtained from the three main components, we made certain changes to the individual variables in the area of the digestive tract to which the formulation components were released. Thus, we found that from each of the investigated preparations, both metals and organic compounds were released into artificial digestive juices; moreover, the release was targeted at a particular place in the digestive tract and depended on the component being analyzed. The organic compounds from the formulations were most released into the intestinal juices. Absorption in the human body is most likely in the intestine. Thus, we conclude that these preparations provide organic compounds to the body. In contrast, the release of metals was in the greatest degree into the gastric juices; this suggests that they are only slightly absorbed by the human body.

## Conclusions

In this study, the usefulness of preparations containing *C. vulgaris* in the supplementation of daily diets with the examined compounds for humans has been evaluated on the basis of the analysis of the extracts incubated with artificial digestive juices, and the concentration of phenolic compounds and lutein in the digestive juices. The qualitative composition of bioelements was consistent with the manufacturer’s declarations on the packaging containing *C. vulgaris*, with respect to the controls, but the examined elements were found to be negligible in the artificial digestive juices. Therefore, these preparations cannot be considered to be a good source of elements such as iron, magnesium, or zinc. An important element in the study of an effect of dietary supplements on humans is also primarily the way of preparing the form of the preparation such that the active substances are released from it in the most effective manner.
